# Adaptive deletion in resistance gene duplications in the malaria vector *Anopheles gambiae*

**DOI:** 10.1111/eva.12619

**Published:** 2018-03-25

**Authors:** Benoît S. Assogba, Haoues Alout, Alphonsine Koffi, Cédric Penetier, Luc S. Djogbénou, Patrick Makoundou, Mylène Weill, Pierrick Labbé

**Affiliations:** ^1^ Institut des Sciences de l'Evolution de Montpellier (UMR 5554, CNRS‐UM‐IRD‐EPHE) Université de Montpellier Montpellier France; ^2^ Disease Control and Elimination Department Medical Research Council, Unit The Gambia Banjul The Gambia; ^3^ Institut Régional de Santé Publique Université d'Abomey Calavi Cotonou Benin; ^4^ Faculté des Sciences et Techniques Laboratoire de Biologie et de Typage Moléculaire en Microbiologie Université d'Abomey Calavi Cotonou Bénin; ^5^ Institut Pierre Richet (IPR)/Institut National de Santé Publique (INSP) Bouaké Côte d'Ivoire; ^6^ Institut de Recherche pour le Développement (IRD) UMR MIVEGEC Montpellier France

**Keywords:** adaptive trajectory, fitness cost, gene duplication, genome evolution, insecticide resistance, malaria vector

## Abstract

While gene copy‐number variations play major roles in long‐term evolution, their early dynamics remains largely unknown. However, examples of their role in short‐term adaptation are accumulating: identical repetitions of a locus (homogeneous duplications) can provide a quantitative advantage, while the association of differing alleles (heterogeneous duplications) allows carrying two functions simultaneously. Such duplications often result from rearrangements of sometimes relatively large chromosome fragments, and even when adaptive, they can be associated with deleterious side effects that should, however, be reduced by subsequent evolution. Here, we took advantage of the unique model provided by the malaria mosquito *Anopheles gambiae s.l*. to investigate the early evolution of several duplications, heterogeneous and homogeneous, segregating in natural populations from West Africa. These duplications encompass ~200 kb and 11 genes, including the adaptive insecticide resistance *ace‐1* locus. Through the survey of several populations from three countries over 3–4 years, we showed that an internal deletion of all coamplified genes except *ace‐1* is currently spreading in West Africa and introgressing from *An. gambiae s.s*. to *An. coluzzii*. Both observations provide evidences of its selection, most likely due to reducing the gene‐dosage disturbances caused by the excessive copies of the nonadaptive genes. Our study thus provides a unique example of the early adaptive trajectory of duplications and underlines the role of the environmental conditions (insecticide treatment practices and species ecology). It also emphasizes the striking diversity of adaptive responses in these mosquitoes and reveals a worrisome process of resistance/cost trade‐off evolution that could impact the control of malaria vectors in Africa.

## 1 INTRODUCTION

The development of new‐generation sequencing technologies (NGS) during the last 15 years enabled empirical measures of the spontaneous rates of mutations in a handful of model organisms. Surprisingly, it revealed that gene duplications and deletions are probably more frequent than substitutions (Katju & Lynch, 2003; Lipinski et al., 2011; Lynch et al., 2008; Schrider, Houle, Lynch, & Hahn, 2013). Copy‐number variations (CNV) are indeed ubiquitous in natural populations (e.g., Freeman et al., 2006). While most of them are probably deleterious (Schrider et al., 2013), they can nonetheless play a crucial role in adaptation and evolution of genome complexity (Assogba et al., 2016; Katju & Bergthorsson, 2013; Kondrashov, 2012; Labbé, Berthomieu et al., 2007; Milesi, Weill, Lenormand, & Labbé, 2017; Schrider & Hahn, 2010).

Two types of gene duplications can be found: (i) homogeneous duplications that result from the amplification of identical copies and (ii) heterogeneous duplications that associate different alleles of the same gene. The quantitative advantage of the first, that is, the increased protein production, is well documented: for example, homogeneous gene duplications have been reported in cases of resistance to insecticides through increased detoxification (Raymond, Chevillon, Guillemaud, Lenormand, & Pasteur, 1998) or in adaptation to a starch‐rich diet in humans and dogs through greater amylase production (Axelsson et al., 2013; Perry et al., 2007). On the contrary, heterogeneous duplications seem to be selected because the two alleles they carry can perform two different functions, by fixing the heterozygote advantage without segregation cost (Haldane, 1932; Milesi, Weill et al., 2017; Spofford, 1969). Such duplications have been documented in a few cases of insecticide resistance, the *Rdl* gene in *Drosophila melanogaster* (Remnant et al., 2013), or the *ace‐1* gene in *Anopheles gambiae* and *Culex pipiens* (Assogba et al., 2016; Labbé, Berthomieu et al., 2007; Milesi, Assogba et al., 2017), where they associate one resistance and one susceptible copy of the gene. While still providing some resistance, this association partially alleviates the deleterious pleiotropic effects (or fitness cost) associated with the resistance allele (Assogba et al., 2015; Labbé et al., 2014; Milesi, Weill et al., 2017).

**Figure 1:**
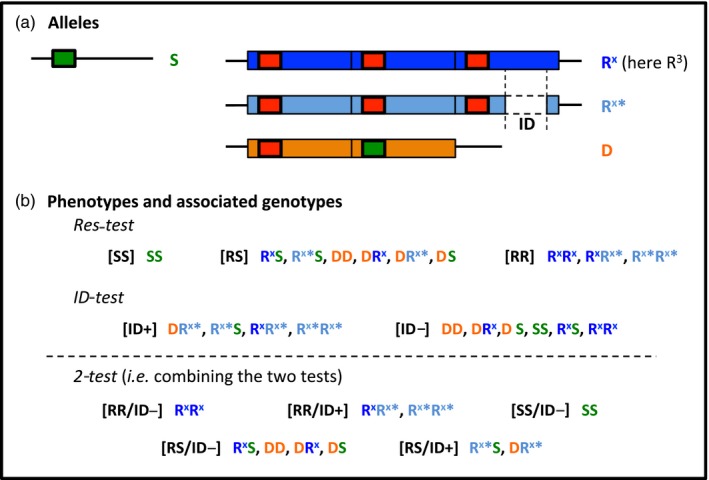
*Anopheles gambiae ace‐1* gene duplicated alleles, genotypes, and phenotypes. (a) The various alleles revealed using the two tests are symbolized: the small boxes represent the *ace‐1* alleles, green for alleles carrying 119G (susceptible), and red for alleles carrying 119S (resistant); the large boxes represent the amplicons (different colors are used to represent the various duplicated alleles although the amplicons are similar as far as we know); the internal deletion (ID) present in one of the amplicons of the R^x^* allele is indicated. (b) For each test (Res‐test or ID test), or the combination of the two (two‐test), the various PCR profiles, *that is,* phenotypes, and associated genotypes are indicated, with conserved color code for each allele. Note that even this combination of tests does not allow complete genotype discrimination

However, duplications are often costly, either through structural problems (breakpoints), hitch‐hiking deleterious mutations, metabolic overproduction costs, and/or due to the disruption of biochemical balances for the products of the duplicated genes (Kondrashov & Kondrashov, 2006; Labbé, Berticat et al., 2007; Milesi, Assogba et al., 2017). The chromosomal segment concerned by the duplication can indeed far exceed the gene of interest so that the resulting amplicons contain several other genes, as shown for example in *Saccharomyces cerevisiae* (Koszul, Caburet, Dujon, & Fischer, 2004), *D. melanogaster* (Remnant et al., 2013), and *An. gambiae* (Assogba et al., 2016). The present study is focused on this latter species, the major malaria vector in Africa, which provides a unique model system to investigate the dynamic and evolution of adaptive duplications: Both homogeneous and heterogeneous duplications of the *ace‐1* gene can be found in this species, providing a large range of adaptive solutions for this mosquito to circumvent insecticide selective pressures (Figure 1a).

The *ace‐1* gene encodes the acetylcholinesterase (AChE1), a synaptic enzyme which is the target of organophosphates (OPs) and carbamates (CXs) insecticides (Massoulié & Bon, 1993). A limited number of single‐base substitutions are responsible for resistance to these insecticide classes: They result in amino acid substitutions in AChE1 that limit the insecticide binding (Alout & Weill, 2008). The G119S substitution (*ace‐1* ^*R*^ allele, or R allele) is the most widespread in natural populations, and it has been selected in several mosquito species (convergent evolution; Weill et al., 2003; Weill, Berthomieu et al., 2004; Weill, Malcolm et al., 2004). In *C. pipiens* and *An. gambiae s.l*., it confers high resistance to CXs and OPs, but has also been shown to decrease the affinity of the resistant enzyme for its substrate by more than 60% relatively to the susceptible enzyme (*ace‐1* ^*S*^ allele, or S allele) (Alout, Djogbénou, Berticat, Chandre, & Weill, 2008; Bourguet, Roig, Toutant, & Arpagaus, 1997). This lower affinity probably underlies the high selective cost of the R allele in both species (Assogba et al., 2015; Berticat, Boquien, Raymond, & Chevillon, 2002; Bourguet, Guillemaud, Chevillon, & Raymond, 2004; Djogbénou, Noel, & Agnew, 2010; Duron et al., 2006; Labbé et al., 2014; Lenormand, Bourguet, Guillemaud, & Raymond, 1999).

A heterogeneous duplication (D allele, Figure 1a) has been found in *An. gambiae s.l*. and associates one S and one R copies (Djogbénou, Chandre et al., 2008). It has recently been shown that this allele provides an intermediate trade‐off, with lower resistance but also lower cost than R, which is probably selected in environments with a mosaic of treated and nontreated areas (Assogba et al., 2015). This D allele appears to be spreading in several West African countries (Djogbénou, Labbé, Chandre, Pasteur, & Weill, 2009).

It has also recently been shown that all *ace‐1* R alleles observed in *An. gambiae s.l*. natural populations actually result from homogeneous duplications containing at least from 2 to 5 R copies (R^x^ alleles, with x between 2 and 5, Figure 1a) (Assogba et al., 2016). The resulting trade‐offs depend on the number of R copies: higher R copy numbers confer higher levels of resistance, but the fitness cost also increases (Assogba et al., 2016).

**Figure 2:**
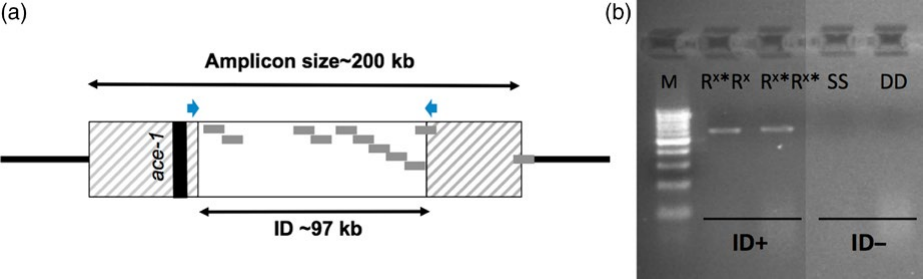
Duplication structure and primer positions of the internal deletion test (ID test). (a) Amplicon structure. The whole amplicon is represented by the box. The predicted genes are represented by gray dots, except for *ace‐1*, which is indicated by the black line (see Assogba et al., 2016 for details). The white box represents the area deleted in some amplicons, that is, the internal deletion (ID). The blue arrows represent the ID test primers positions. (b) PCR results of the ID test for different genotypes (NB: This image has been produced by merging two parts of a single photograph). Only those containing an R^x^* copy are amplified ([ID+]). M is the size marker

What is the cause of this increased cost? A hint at the answer came from NGS analyses that allowed deciphering the *ace‐1* duplication genomic structure (Assogba et al., 2016). In both homogeneous and heterogenous duplications, the amplicon borders are strictly identical, to the base: they consist of ~200 kb chromosome fragments containing *ace‐1*, but also ten other genes. However, an internal deletion (ID) was identified in one of the amplicons of the three‐copies homogeneous duplication (R^3^*) found in the laboratory strain AcerkisR^3^ (Figures 1 and 2); this ID was also found in genomic data from natural vector populations collected in Burkina Faso and Guinea (Assogba et al., 2016). Curiously, this deletion removes all the amplified genes, but *ace‐1*,* that is,* in a R^3^* allele, there are three copies of *ace‐1*, but only two copies of the ten other genes, as in D alleles (Figure 2).

The current hypothesis is thus that the cost of the homogeneous duplications is probably related to gene‐dosage imbalance; postduplication internal deletions can then be selected because they reduce these protein overdoses. To test this hypothesis, we developed a diagnostic PCR test to detect the deletion in *An. gambiae s.l*. and screened seven field populations (1086 individuals) collected over several years from three West African countries (Benin, Togo, and Ivory Coast). This large survey revealed that the internal deletion is recurrent and pervasive, and supports the hypothesis that it reduces the fitness cost associated with R^x^ allele homogeneous duplications. This adaptive trajectory in response to changing environment selection pressures, and its consequences for current resistance and malaria management are discussed.

## 2 MATERIALS AND METHODS

### 2.1 Mosquito collections

Larvae from seven *An. gambiae s.l*. field populations were collected and reared until adulthood in the laboratory: one from Benin, one from Togo, and five from Ivory Coast; each was sampled two to four times (19 samples in total, Table 1). Adults were assigned to members of the *An. gambiae* cryptic‐species complex on the basis of morphological tests and molecular analyses (Gillies & Coetzee, 1987; Santolamazza et al., 2008; Scott, Brogdon, & Collins, 1993).

**Table 1:**
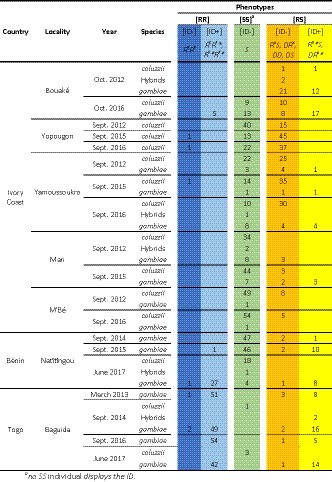
Phenotyping results. The first four columns give the country, locality, and year of collection each sample. For each collection, and the three categories of mosquitoes (*An. gambiae s.s*., *An. coluzzii,* and their hybrids) are given the numbers of each phenotype identified by Res‐test ([RR], [SS], and [RS]) and ID test ([ID‐] and [ID+]). The genotypes corresponding to the combination of the two molecular tests are indicated in italics (see text and Figure 1 for a summary of the resulting phenotypes). The colors refer to the different alleles, as in Figures 1 and 4

### 2.2 Specific molecular tests

#### 2.2.1 ace‐1 resistance phenotype (Res‐test)

The *ace‐1* (AGAP001356, https://www.vectorbase.org/) resistance phenotype (susceptible individuals [SS] with only S copies, homozygous‐resistant individuals [RR] with only R copies, or heterozygous individuals [RS] with both S and R copies) was assessed for each individual using the *ace‐1* PCR‐RFLP test developed by Weill, Malcolm et al. (2004). We refer to phenotypes rather than genotypes for the different profiles resulting from the PCR, because they do not allow discriminating the various genotypes (i.e., duplicated allele vs. standard heterozygotes, Figure 1a), as well as the number of *ace‐1* copies.

#### 2.2.2 Diagnostic PCR test for the 97‐kb internal deletion (ID test)

A PCR primer pair was designed (Del97dir1 and Del97rev2) with each primer sitting on either side of the ID (Table S1 and Figure 2). The resulting 584‐bp fragment overlaps the ID region; it is amplified only in individuals carrying this specific deletion, the fragment lacking deletion being too long for PCR amplification. This rapid diagnostic PCR test is dominant and reveals the presence of the ID when present in at least one amplicon. It results in two PCR profiles, or phenotypes, [ID+] or [ID‐], respectively, for individuals carrying at least one ID (R^x^* allele) or none at all (R^x^ allele).

### 2.3 Gene copy‐number quantification

We estimated the relative number of copies present for two target regions, the *ace‐1* locus and the region overlapping the ID by real‐time quantitative PCR (qPCR, LC480 LightCycler^®^, Roche). We used the Rps7 locus, present as a single copy in the VectorBase PEST genome (AGAP010592; https://www.vectorbase.org/), as reference (AgS7Ex5qtidir and AgS7Ex5qtirev primers were used to amplify a 107‐bp fragment, Table S1). We used the primer pair AgAce1qtidir2 and AgAce1qtirev2 primers to amplify a 185‐bp fragment of the *ace‐1* gene (Table S1), and the primer pair Del97Qdir5 and Del97Qrev4 to amplify a 186‐bp fragment overlapping the ID (Table S1).

We used the qPCR amplification conditions described by Assogba et al. (2016): 0.5 μl of genomic DNA and 1.5 μl of reaction mix containing 0.8 μM of each specific primer and 0.75 μl of mastermix (LightCycler^®^ 480 SYBR Green I Master, Roche) were dispensed on a 384‐well plate using the Labcyte^®^ Echo525 dispenser. The qPCR was performed with a 95°C activation step for 8 min followed by 45 cycles of 95°C for 4‐s, 67°C for 13 s, and 72°C for 19 s. Melting curves were generated by a postamplification melting step between 70°C and 95°C, for Tm analysis. All quantifications were replicated four times for each DNA template. Standard curves were constructed using 10 to 10 dilutions of a PCR product previously amplified: (i) on KisumuP (SS) strain DNA for *ace‐1* and *RpS7* specific primers and (ii) on AcerkisR^3^ (R^3^*R^3^*) strain DNA for the ID region‐specific primers (this strain present a single ID on one of the three *ace‐1*‐encompassing amplicons carried by each chromosome, Assogba et al., 2016). *ace‐1* and ID copy‐number ratios over *RpS7* were determined using the advanced relative quantification method (LightCycler^®^ 480 software v.1.5.0).

We confirmed the real‐time qPCR results using droplet digital PCR or ddPCR (Vogelstein & Kinzler, 1999), in particular for the individuals presenting the highest levels of amplification, as this second approach is more reliable in these conditions. For the ddPCR assay, 10 ng of DNA was assayed in a final volume of 20 μl containing 1× ddPCR EvaGreen^®^ supermix and 0.1 μM of each primer (Del97Qdir5 and Del97Qrev4, Table S1). Droplets were generated from this PCR mix using a eight‐channel droplet generator cartridge, transferred to a 96‐well plate, and then amplified using a thermal cycler, according to manufacturer recommendations (Bio‐Rad). Thermal cycling conditions were as follows: 95 °C for 10 min, 94 °C for 30 sec and 60 °C for 1 min (40 cycles), and 98 °C for 10 min. After PCR amplification, the cycled droplets were read individually with the QX200 droplet reader (Bio‐Rad) and analyzed with QuantaSoft^®^ droplet reader software, version 1.6.6.0320 (Bio‐Rad).

### 2.4 Statistical analyses

#### 2.4.1 Copy‐number dynamics

The numbers of *ace‐1* and ID copies were quantified for 3 years (2013, 2014, and 2016) in Baguida (Togo). The significance of the observed differences was assessed with the following generalized linear model (GLM): CN = YEAR + ε, where CN is the number of copies for each individual, YEAR is a three‐level factor corresponding to the year of sampling, and ε is the error parameter, which follows a Gaussian distribution. The significance of the YEAR effect was tested using a likelihood‐ratio test (LRT) between the full model and a model without this effect; years that were not significantly different (LRT) were grouped (Crawley, 2007). We checked the normality of the model residuals and homoscedasticity using Shapiro–Wilk and Breusch–Pagan tests, respectively. All computations were performed using the R free software (v.3.3.1, http://www.r-project.org, The R core Team).

#### 2.4.2 Allele frequencies

As the tests used only partially discriminate the various genotypes, allele frequencies cannot be calculated directly. Instead, they were estimated from the phenotypes (as defined by the combined PCR profiles in Res‐test and ID test, see Figure 1b, two‐test), assuming panmixia and independently for each locality and each year, using the maximum‐likelihood approach developed by Lenormand, Guillemaud, Bourguet, and Raymond (1998).

Briefly, we calculated the log‐likelihood *L* of observing all the data:

$$L=\sum_{i}n_{\mathrm{ijt}}\text{ln}\left(f_{\mathrm{ijt}}\right),$$

with *n*_*ijt*_ and *f*_*ijt,*_ respectively, the observed number and the predicted frequency of individuals with phenotype *i* in population *j* at time *t*. It was simultaneously maximized (*L*_max_) for each sample using a simulated annealing algorithm (Labbé, Sidos, Raymond, & Lenormand, 2009; Lenormand, Guillemaud, Bourguet, & Raymond, 1998; Milesi, Lenormand, Lagneau, Weill, & Labbé, 2016). For each allele frequency, the support limits (SL) were calculated as the minimum and maximum values that it could take without significantly decreasing the likelihood (Labbé et al., 2009; Milesi et al., 2016); SL are roughly equivalent to 95% confidence intervals. Recursions and likelihood maximization algorithms were written and compiled with Lazarus v1.0.10 (http://www.lazarus.freepascal.org/).

## 3 RESULTS

### 3.1 The internal deletion is pervasive in RR individuals from *An. gambiae* field populations

Characterizing the genomic structure of the *ace‐1* homogeneous duplication in the R^3^R^3^‐resistant strain (AcerKisR^3^) revealed a 97‐kb internal deletion (ID) in one of its three amplicons (Assogba et al., 2016). Alleles displaying this ID (whether in one or several amplicons) will thereafter be called R^x^*, while those without the ID will be called R^x^ (S alleles should not display the ID as [SS] individuals were shown negative for the *ace‐1* homogenous duplication; Assogba et al., 2016). To understand the adaptive role of this ID, we developed a diagnostic PCR test (ID test) to study *An. gambiae s.l*. field populations. As the PCR primers sit on either side of the deletion (Figure 2 and Table S1), a positive amplification (phenotype [ID+], corresponding to the allele R^x^*) should occur only when this specific ID is present in at least one amplicon.

We first validated the ID test on the reference susceptible (KisumuP, Shute, 1956) strain and on a reference strain carrying the heterogeneous duplicated D allele (AcerdupliKis, Assogba et al., 2015), which proved to be both [ID‐] as expected. We then screened 19 field populations of *An. gambiae s.l*. collected in Benin, Togo, and Ivory Coast over several years (Table 1). All mosquitoes were first typed using the Res‐test, which discriminates [SS], [RS], and [RR] phenotypes (Weill, Malcolm et al., 2004), then using the ID test. The 476 [SS] field individuals were [ID‐], while 229 (97%) of the 236 [RR] individuals were [ID+]. This result confirms the specificity of the ID test and shows that the internal deletion (ID) is extremely frequent in field populations ([RR] individuals without at least one ID were extremely rare).

**Figure 3:**
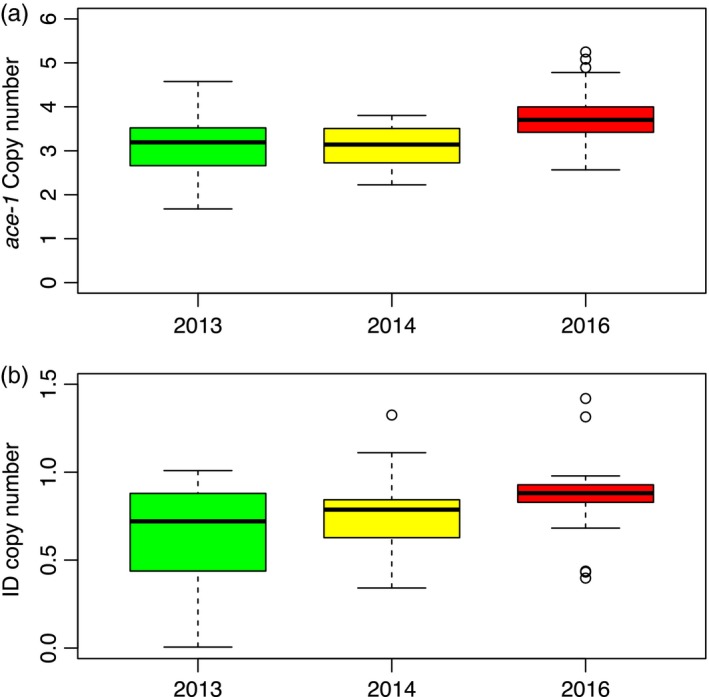
Evolution of the number of *ace‐1* and ID copies in [RR] individuals from Baguida (Togo). Box plot represents the distributions of the copy numbers ([a] *ace‐1*, [b] ID) in individuals sampled in Baguida in 2013, 2014, and 2016. The bold line represents the median, the box and whiskers, respectively, represent the 25% and 75%, and 5% and 95% quartiles, and the dots represent outliers

We further investigated the highly frequent [RR] individuals of the Baguida population (Togo, Table 1) to analyze the relative proportion of *ace‐1* gene copies carrying or not an internal deletion and their dynamics (Figure 3 and Table S2). We used R^3^*R^3^* individuals as reference (AcerKisR^3^ strain): they carry two R* copies and four R copies. As their copy number is expressed relatively to the Rps7 locus, present in two copies per genome, these R^3^*R^3^* individuals display a relative *ace‐1* copy number of 3 (6/2) and a relative ID copy number of 1 (2/2; Figure 1a; Assogba et al., 2016). Similarly, R^3^R^3^ individuals (no ID) would display a *ace‐1* copy number of 3 (6/2) and a ID copy number of 0 (0/2), while R^3^R^3^* (one ID only) individuals would display a *ace‐1* copy number of 3 (6/2) and a ID copy number of 0.5 (1/2; Figure 1a). In the Baguida population, we found a significant increase in *ace‐1* copy number between samples collected in 2013 or 2014 (respectively, 3.11 ± 0.61 and 3.12 ± 0.44, GLM, LRT, *F *= 0.003*, p *=* *.95) and samples collected in 2016 (3.77 ± 0.61; GLM, LRT, *F *=* *17.8*, p *<* *.001; Figure 3a). Only four individuals marginally exceeded an ID copy number of 1, which suggests that most resistance alleles carry at best one ID in one of their amplicons. Moreover, the ID copy number significantly increased over the years, from 0.66 ± 0.25 in 2013, to 0.76 ± 0.22 in 2014, and to 0.85 ± 0.19 in 2016 (GLM, LRT, *F* = 7.6, *p *<* *.001; Figure 3b). This suggests that, while a high proportion of the [RR] individuals were probably of genotype R^x^R^x^* (i.e., ID copy number = 0.5) in 2013, most were R^x^*R^x^* in 2016 (i.e., ID copy number = 1). Note that only 3 R^x^R^x^ individuals ([ID‐]) were found of 199 [RR] in Baguida, one in 2013, two in 2014, but none in 2016 (Table 1).

### 3.2 Allele frequencies are different between populations and species

Four alleles (or allele classes) were segregating in the studied field populations of *An. gambiae s.s* and *Anopheles coluzzii*: R^x^ (the resistant allele without ID), R^x^* (the resistant allele with ID), D (the heterogeneous duplication), and S (the susceptible allele); their combinations thus result in 10 possible genotypes (Figure 1). However, combining the Res‐test and the ID test allows the discrimination of only five PCR profiles, that is, the two‐test phenotypes (Figure 1b). In particular, standard heterozygotes (R^x^S or R^x^*S) cannot be differentiated from D‐carriers (DD, DS, DR^x^, or DR^x^*).

**Figure 4:**
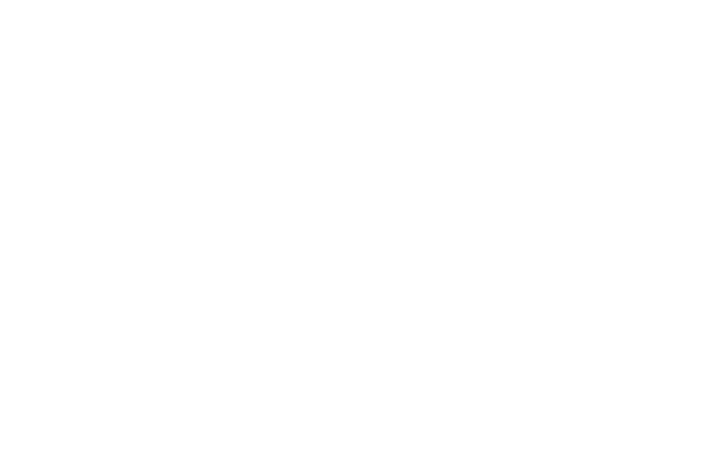
Allele frequencies. The cumulated frequencies of the R^x^, R^x^*, D, and S alleles are presented for each sample. The locality and year of collection are also indicated (bottom), as well as the number of analyzed individuals (*N*) and the species (top). Note that only samples with more than 10 individuals were considered to estimate the allelic frequencies using the maximum‐likelihood approach (see text and Table S3). Colors are the same than in Figure 1

Consequently, we used a maximum‐likelihood approach to estimate, in each sample, the frequencies of the four alleles from the number of individuals in each of the two‐test phenotypes (Figure 4 and Table S3). We first observed strong differences between the two species, with a significantly higher global resistance frequency in *An. gambiae s.s*. relatively to *An. coluzzii*: Mean cumulated resistance allele frequency (i.e., *f*_*D*_ + *f*_*R*_ + *f*_Rx*_) was equal to 0.59 ± 0.41 and 0.23 ± 0.21, respectively (Welch *t* test, *t*_16.27_ = 2.66, *p *=* *.017). Moreover, the cumulated resistance frequencies were very variable between populations in both species (from 0 to 0.53 in *An. coluzzii* and from 0.03 to 1 in *An. gambiae s.s*., Figure 4 and Table S3).

The frequencies of the different resistance alleles (R^x^, R^x^*, D) appeared very variable between localities and species (Figure 4 and Table S3). Thus, R^x^* was globally more frequent than R^x^, with a sharp difference between *An. Coluzzii* (in which R^x^* was almost absent) and *An. gambiae s.s* (in which R^x^* was generally the most frequent resistance allele). Overall, D was present in most populations: in *An. coluzzii*, its frequency was higher than that of R^x^, reaching 0.5 in some populations; in *An. gambiae s.s*., D had generally a low frequency (except in 2012 in Bouaké) and R^x^ was rarely found (Figure 4 and Table S3).

Note that the field populations screening revealed 374 [RS] individuals, among which 271 were [ID‐] and 103 [ID+] (27.5%, Table 1). In populations displaying a large excess of heterozygotes (Bouaké, Yopougon, and Yamoussoukro), thus with a high frequency of D alleles (see Lenormand, Guillemaud et al., 1998), the frequency of R^x^* is generally limited (Figure 4 and Table S3). This is in agreement with the previous observation indicating that the D allele does not carry an internal deletion similar to that found in R^x^* (Assogba et al., 2016): [RS/ID+] individuals are either DR^x^* or R^x^*S individuals.

Considering the temporal variations, while some populations appeared quite stable (e.g., Yamoussouko, Man or M'bé), others displayed strong fluctuations between years (Figure 4). For example, resistance increased sharply in Natintingou (*An. gambiae s.s*.) and in Yopougon (*An. coluzzii*); on the contrary, it decreased in Bouaké (*An. gambiae s.s*.). Moreover, there were variations in the relative frequencies of the resistance alleles: In Bouaké (*An. gambiae s.s*.), there was a sharp reduction in D allele frequency, R^x^* allele becoming the most frequent resistance allele; in Baguida (*An. gambiae s.s*.), while no susceptible individual was found over 4 years, the R^x^ allele appeared almost eliminated by the R^x^* allele (Figure 4).

## 4 DISCUSSION

The ~200‐kb homogeneous duplication surrounding the *ace‐1* gene in *An. gambiae* mosquitoes contains 10 other genes; an internal deletion (ID) eliminating these 10 genes in one of the three amplicons of the AcerKisR^3^ strain was also discovered (Assogba et al., 2016). In this study, we tested whether the ID found in the *ace‐1* homogeneous duplications were indeed adaptive and selected in natural populations of *An. gambiae s.l*.

### 4.1 The internal deletion is spreading in West Africa and between *Anopheles* species

We first assessed the distribution of the ID in several populations of *An. gambiae s.l*. in three adjacent countries of West Africa (Ivory Coast, Benin, and Togo). We designed a specific molecular test (ID test), which we combined to the classic *ace‐1* resistance test (Res‐test, Weill, Malcolm et al., 2004). This ID test amplifies a fragment only when the deletion is present (Figure 2), it is thus highly specific: When positive it is the very same deleted allele that is detected, R^x^*, as it is very unlikely that this particular deletion event (same size, same breaking points) occurred more than once.

We first demonstrated that the ID is only found in homogeneous duplications: indeed, no S alleles were amplified and we found no evidence of its presence in heterogeneous duplications (D alleles) (Table 1). However, we found the R^x^* allele in all countries sampled in the present study, and the same ID was detected previously from genomic analysis in mosquitoes from Burkina Faso and Guinea sequenced by the *An. gambiae* 1,000 Genomes Consortium (Assogba et al., 2016). These results thus confirm the pervasive character of this ID in most of West African *An*. *gambiae s.l*. populations.

However, inferring the frequency of the four alleles segregating in these populations (R^x^, R^x^*, D, and S) revealed strong variations between populations and between species (Figure 4). In particular, R^x^* was found at high frequencies in most *An. gambiae s.s*. populations, whereas it was nearly absent from *An. coluzzii*, even in localities where both species coexists (Natitingou 2017, Bouaké 2016 and Yamoussoukro 2016, Figure 4). This suggests that the deletion may have occurred first in *An. gambiae s.s*. and recently introgressed in *An. coluzzii*: we found a few hybrids, some carrying a R^x^* allele (as in Baguida 2014, Togo; Table 1), supporting this hypothesis. Note that the *ace‐1* R and the *ace‐1* D alleles similarly spread between *An. gambiae s.s*. and *An. coluzzii* through introgression (Djogbénou, Chandre et al., 2008).

### 4.2 The internal deletion is adaptive

This large distribution alone suggests that the ID is adaptive. This hypothesis is nevertheless strengthened by several evidences from R^x^* intrapopulation dynamics. First, the R^x^ (the resistance allele without ID) was much less frequent than R^x^* (Figure 4), and among the [RR] phenotypes (resistant homozygotes), very few R^x^R^x^ were identified (2.96%, Table 1), which suggests a higher cost of R^x^ when homozygous than R^x^*. Second, over the few years of survey, several populations showed either a faster increase of R^x^* than of R^x^, in a general context of increasing resistance (Bouaké, Ivory Coast, or Natitingou, Benin), or even the elimination of R^x^ by R^x^* (Baguida, Togo, Figure 4). Finally, we simultaneously measured in [RR] individuals from Baguida both the number of *ace‐1* copies and the number of amplicons affected by the ID (Figure 3). We found that homogeneous duplications carried three or more *ace‐1* R copies, but that only one amplicon was affected by the ID. More importantly, during the 3 years of survey, [RR] individuals were mostly R^x^R^x^* at the beginning and became more and more homozygotes R^x^*R^x^* in the following years (the mean ID copy number increasing from 0.66 to 0.85, Figure 3). This confirms the rapid replacement of R^x^ by R^x^* in this population (Figure 4).

Previous work showed that a higher number of *ace‐1*‐resistant copies resulted in a higher fitness cost to its carriers, but also higher resistance levels (Assogba et al., 2016). As the deletion does not affect the *ace‐1* locus (Figure 2), it should not affect the resistance level (i.e., R^x^ should be as resistant as R^x^*). All the previous observations thus indicate that R^x^* is selected over R^x^ because it is less costly. Similar to resistance, the cost reduction induced by the ID does not affect *ace‐1* and most probably results from the partial restoration of gene‐dosage balance in coamplified loci (as the ~200 kb amplicon encompasses 10 other genes; Assogba et al., 2016). The increased gene dosage of the coamplified loci could indeed (i) alter biochemical equilibria between duplicated and nonduplicated interacting genes (Birchler & Veitia, 2007; Papp, Pal, & Hurst, 2003), (ii) overshoot optimal protein levels, thereby altering their function (Conrad & Antonarakis, 2007; Lupski et al., 1992), or (iii) increase the energy required for their production (Kalisky, Dekel, & Alon, 2007), all costs that may combine. Postduplication genomic rearrangements reducing the cost of gene‐dosage disturbance (such as the deletion studied here) are thus expected to be selected. Interestingly, as i) D alleles did not carry the ID (but carry two copies of these 10 genes), and ii) ID affected only one amplicon in R^3^, it suggests that the gene‐dosage cost probably becomes a significant hindrance over 2 copies.

### 4.3 Insecticide treatment practices are heterogeneous in West Africa and affect the nature of the selected resistance allele

In *An. gambiae*, the heterogeneous duplicated allele D has been shown to confer intermediate resistance level as well as intermediate fitness cost, similar to standard R^x^S heterozygotes (Assogba et al., 2015). This allele is thus favored in areas where the selective pressure is moderate or in heterogeneous environments, with mosaic of treated and nontreated areas and/or discontinuous application of insecticides. On the contrary, homogeneous duplicated alleles R^x^ have been shown to be more resistant and more costly than D alleles; moreover, R^x^ alleles confer increased resistance, and cost, when the number of R copies increase (Assogba et al., 2016). These alleles are thus favored in highly treated areas.

Our survey suggests that treatment practices could differ substantially between the different collection sites: resistant allele frequencies were globally higher in Baguida, Togo (where almost no S allele was found), probably reflecting more intense insecticide treatments than in populations sampled in Ivory Coast and Benin (Table 1 and Figure 4). Accordingly, while the D allele prevailed in most Ivory Coast populations (for both species), R^x^* was the predominant allele in *An. gambiae s.s* from Baguida (Table 1 and Figure 4). Resistance frequencies, and thus probably treatment intensities, appeared globally stable over time, except in *An. gambiae s.s* from Natitingou (Benin), where a sharp increase was observed in 2017 that resulted in a surge in R^x^* frequency (Figure 4; this area of Benin is treated using indoor residual spreading since 2012 as part of the President's Malaria Initiative, PMI, http://www.africairs.net/where-we-work/benin/).

There are also sharp contrasts between species, as *An. gambiae s.s*. is globally more resistant than *An. coluzzii* (Table 1 and Figure 4), an observation consistent with previous reports from several West African countries (Dabiré et al., 2009; Djogbénou, Akogbéto, & Chandre, 2008; Djogbénou, Chandre et al., 2008; Djogbénou et al., 2009; Essandoh, Yawson, & Weetman, 2013; Weetman et al., 2015), although not all (see Ahoua Alou et al., 2010; Koffi, Ahoua Alou, Adja, Chandre, & Pennetier, 2013 for data from Ivory Coast localities). These differences can be particularly striking in samples where both species coexist: for example, in Natitingou 2017, the frequency of resistance alleles is about 0.8 in *An. gambiae s.s*., but 0 in *An. coluzzii* (Figure 4). Complementing these observations, the predominant resistance allele in *An. coluzzii* is D (intermediate resistance/intermediate cost), while it is R^x^* (high resistance/high cost) in *An. gambiae s.s*. (Figure 4 and Table S3). Together, these findings suggest contrasted exposures to insecticides, *An. gambiae s.s*. being exposed to higher insecticide doses and/or more homogeneous treatments in space and/or time than *An. coluzzii*. These differences would probably be the result of habitat preferences: in West Africa, *An. coluzzii* is colonizing arid areas with permanent large breeding sites, while *An. gambiae s.s*. prefers wetter areas, with small and ephemeral water bodies (Lehmann, Diabate, & Diabaté, 2008); species preferences seem different in forests of Central Africa (Kamdem et al., 2012). Dabiré et al. (2009) nonetheless suggested that *An. gambiae s.s*. was more exposed to agricultural insecticides than its sibling species. One possibility is that, due to the limited size of its breeding sites, *An. gambiae s.s*. is usually exposed to higher doses of insecticides, resulting in higher and more constant selective pressures than *An. coluzzii,* for which large natural habitats would result in exposure to more diluted and more variable insecticide doses. Similarly, at the adult stage, *An. coluzzii* has been shown to be more exophilic and exophagic than *An. gambiae s.s*. (Moiroux et al., 2014) and is thus less exposed to indoor insecticide treatments (indoor residual spraying or treated bed‐nets).

Unfortunately, the high heterogeneity and limited oversight in treatment practices in the considered countries make it difficult to directly relate them with resistance. However, our study suggests that both resistance frequency and the nature of the selected resistance alleles directly depend on the insecticide treatment regimens.

In conclusion, our study provides a unique example of a postduplication modification that increased the fitness of an adaptive duplication: a single deletion partly reduces the gene‐dosage disturbances in nonadaptive genes picked up in the large amplicon containing the adaptive locus. Thus, while a duplication event often causes major genome disturbances, these can be alleviated by further evolution, provided that the selective advantage of the original duplication is high enough.

From a more applied point of view however, this fascinating variety of duplications, both heterogeneous and homogeneous, provides *An. gambiae* with a large adaptive capacity to various treatment regimens. Unfortunately, resistance (and particularly *ace‐1* R alleles) has been shown to impact the malaria pathogen transmission (Alout et al., 2013; Alout, Djègbè et al., 2014; Alout, Yameogo et al., 2014; Alout et al., 2016), although its net impact on malaria transmission is still debated (Alout, Labbé, Chandre, & Cohuet, 2017). The original finding, in R^x^ alleles, of a cost proportionally increasing with the R copy number, suggested a reassuring cap to the levels of resistance reachable by *An. gambiae s.l*. mosquitoes; however, a postduplication deletion is now spreading in natural populations, and between species, because it alleviates this cost, which is more worrisome. It makes the resistance/cost trade‐off of these alleles more favorable to the mosquitoes and may have a major impact on the control of this major malaria vector in Africa.

## DATA ARCHIVING

Data for this study are all available in the main document or supplementary material.

## CONFLICT OF INTEREST

None declared.
