# Supplementary material for: Adaptive deletion in resistance gene duplications in the malaria vector Anopheles gambiae
Source: Evol Appl. 2018 Mar 25;11(8):1245–56. doi: 10.1111/eva.12619 (PMC6099818; doi:10.1111/eva.12619)
Supplement: Supplementary file 1 [file EVA-11-1245-s001.docx]

| **Marker name** | **Locus** | **Chromosome** | **Primer (5’-3’)** | **Size**  **(bp)** | **Tm**  **(°C)** |
| --- | --- | --- | --- | --- | --- |
| *ace-1 resistance phenotype (Res-test)* | AGAP001356  (*ace-1)* | 2R | Exon3univdir: GATCGTGGACACCGTGTTCG  Exon3univrev: AGGATGGCCCGCTGGAACAG | 541 | 58 |
| *Internal Deletion test*  *(ID-test)* | AGAP001357 AGAP001367 | 2R | Del97dir1: AGCGTAAGCGCACGAGGAA  Del97rev2: CATCTTCCCGCAACAGTGT | 584 | 58 |
| *qPCR* | AGAP001356  (*ace-1)* | 2R | AgAce1qtidir2 : ATGTGGAACCCGAACACG  AgAce1qtirev2 : ACCACGATCACGTTCTCCTC | 185 | 67 |
|  | AGAP010592  (*RpS7*, control) | 3L | AgS7Ex5qtidir : GTGTACAAGAAGCTGACTGGC  AgS7Ex5qtirev : TAGCTGCTGCAAACTTCGG | 107 | 67 |
|  | AGAP001357  AGAP001367 | 2R | Del97Q dir5 : GAAAGCGAGATTGAGCTGAA  Del97Qrev4 : GGGTTAGTACATTTCTCGCG | 186 | 67 |

**Table S1: List of the primers used in this study**

**Table S2: Raw data of the number of *ace-1* and ID copies in [RR] individuals from Baguida (Togo).** The qPCR or ddPCR values (after standardization, see Material and Methods) for *ace-1* and for ID are given for each individual (Ind.) and each year (Year).

| **Ind.** | **Year** | ***ace-1* copy number** | **ID copy number** |  | **Ind.** | **Year** | ***ace-1* copy number** | **ID copy number** |  | **Ind.** | **Year** | ***ace-1* copy number** | **ID copy number** |
| --- | --- | --- | --- | --- | --- | --- | --- | --- | --- | --- | --- | --- | --- |
| 1 | 2013 | 4.576 | 1.009 |  | 1 | 2014 | 2.880 | 0.516 |  | 1 | 2016 | 3.641 | 0.834 |
| 2 | 2013 | 3.604 | 0.879 |  | 2 | 2014 | 2.677 | 0.829 |  | 2 | 2016 | 3.759 | 0.825 |
| 3 | 2013 | 2.350 | 0.607 |  | 3 | 2014 | 2.885 | 0.610 |  | 3 | 2016 | 3.858 | 0.835 |
| 4 | 2013 | 2.495 | 0.467 |  | 4 | 2014 | 3.255 | 0.840 |  | 4 | 2016 | 3.075 | 0.432 |
| 5 | 2013 | 2.626 | 0.438 |  | 5 | 2014 | 2.225 | 0.437 |  | 5 | 2016 | 3.548 | 0.906 |
| 6 | 2013 | 3.087 | 0.512 |  | 6 | 2014 | 3.507 | 0.993 |  | 6 | 2016 | 3.792 | 1.419 |
| 7 | 2013 | 2.603 | 0.452 |  | 7 | 2014 | 3.347 | 0.814 |  | 7 | 2016 | 3.042 | 0.781 |
| 8 | 2013 | 3.423 | 0.870 |  | 8 | 2014 | 3.337 | 1.025 |  | 8 | 2016 | 2.581 | 0.398 |
| 9 | 2013 | 2.878 | 0.721 |  | 9 | 2014 | 3.198 | 0.928 |  | 9 | 2016 | 3.356 | 0.838 |
| 10 | 2013 | 1.874 | 0.463 |  | 10 | 2014 | 2.945 | 0.924 |  | 10 | 2016 | 3.694 | 0.942 |
| 11 | 2013 | 3.444 | 0.923 |  | 11 | 2014 | 3.085 | 1.111 |  | 11 | 2016 | 4.892 | 0.932 |
| 12 | 2013 | 3.958 | 0.920 |  | 12 | 2014 | 2.726 | 0.566 |  | 12 | 2016 | 3.545 | 0.928 |
| 13 | 2013 | 2.868 | 0.741 |  | 13 | 2014 | 3.622 | 1.057 |  | 13 | 2016 | 3.578 | 0.876 |
| 14 | 2013 | 1.678 | 0.006 |  | 14 | 2014 | 3.018 | 1.064 |  | 14 | 2016 | 4.594 | 0.935 |
| 15 | 2013 | 4.184 | 0.928 |  | 15 | 2014 | 3.052 | 0.843 |  | 15 | 2016 | 3.627 | 0.902 |
| 16 | 2013 | 3.133 | 0.387 |  | 16 | 2014 | 3.802 | 1.325 |  | 16 | 2016 | 3.973 | 0.865 |
| 17 | 2013 | 3.322 | 0.869 |  | 17 | 2014 | 2.692 | 0.889 |  | 17 | 2016 | 3.096 | 0.922 |
| 18 | 2013 | 3.243 | 0.413 |  | 18 | 2014 | 2.393 | 0.818 |  | 18 | 2016 | 3.650 | 0.927 |
| 19 | 2013 | 2.661 | 0.407 |  | 19 | 2014 | 3.579 | 0.628 |  | 19 | 2016 | 2.567 | 0.437 |
| 20 | 2013 | 2.958 | 0.489 |  | 20 | 2014 | 3.591 | 0.820 |  | 20 | 2016 | 4.002 | 0.929 |
| 21 | 2013 | 3.542 | 0.461 |  | 21 | 2014 | 2.886 | 0.674 |  | 21 | 2016 | 4.782 | 0.682 |
| 22 | 2013 | 3.522 | 0.855 |  | 22 | 2014 | 3.583 | 0.800 |  | 22 | 2016 | 5.248 | 0.892 |
| 23 | 2013 | 3.664 | 0.532 |  | 23 | 2014 | 2.963 | 0.587 |  | 23 | 2016 | 3.340 | 0.899 |
| 24 | 2013 | 2.634 | 0.723 |  | 24 | 2014 | 3.704 | 0.756 |  | 24 | 2016 | 3.594 | 0.701 |
| 25 | 2013 | 3.274 | 0.920 |  | 25 | 2014 | 3.326 | 0.658 |  | 25 | 2016 | 3.322 | 0.843 |
| 26 | 2013 | 2.442 | 0.416 |  | 26 | 2014 | 2.401 | 0.656 |  | 26 | 2016 | 4.387 | 0.973 |
| 27 | 2013 | 2.272 | 0.346 |  | 27 | 2014 | 3.327 | 0.403 |  | 27 | 2016 | 3.911 | 0.977 |
| 28 | 2013 | 3.528 | 0.845 |  | 28 | 2014 | 2.534 | 0.342 |  | 28 | 2016 | 4.018 | 0.979 |
| 29 | 2013 | 2.741 | 0.402 |  | 29 | 2014 | 3.702 | 0.783 |  | 29 | 2016 | 3.836 | 0.885 |
| 30 | 2013 | 3.267 | 0.432 |  | 30 | 2014 | 3.667 | 0.831 |  | 30 | 2016 | 3.703 | 0.833 |
| 31 | 2013 | 3.182 | 0.854 |  | 31 | 2014 | 3.314 | 0.703 |  | 31 | 2016 | 4.252 | 0.769 |
| 32 | 2013 | 3.192 | 0.391 |  | 32 | 2014 | 3.681 | 0.782 |  | 32 | 2016 | 4.429 | 0.856 |
| 33 | 2013 | 3.657 | 0.977 |  | 33 | 2014 | 2.451 | 0.351 |  | 33 | 2016 | 3.487 | 0.792 |
| 34 | 2013 | 3.892 | 0.993 |  | 34 | 2014 | 2.644 | 0.697 |  | 34 | 2016 | 3.704 | 0.877 |
| 35 | 2013 | 3.200 | 0.967 |  | 35 | 2014 | 3.397 | 0.795 |  | 35 | 2016 | 2.901 | 0.436 |
| 36 | 2013 | 2.726 | 0.797 |  | 36 | 2014 | 2.666 | 0.391 |  | 36 | 2016 | 3.860 | 0.920 |
| 37 | 2013 | 3.351 | 0.904 |  | 37 | 2014 | 3.325 | 0.792 |  | 37 | 2016 | 5.084 | 1.315 |
|  |  |  |  |  | 38 | 2014 | 3.057 | 0.738 |  | 38 | 2016 | 3.098 | 0.948 |
|  |  |  |  |  |  |  |  |  |  | 39 | 2016 | 3.856 | 0.861 |
|  |  |  |  |  |  |  |  |  |  | 40 | 2016 | 3.995 | 0.895 |

**Table S3: Allele frequencies estimations at the *ace-1* locus.** For each sample, the country, locality and year of collection are indicated, as well as the frequencies of the various alleles estimated from the *2-test* phenotypes (Tab. 1) using a maximum likelihood approach; the support limits (minimum and maximum, roughly equivalent to 95% confidence intervals) are indicated in brackets. A "-" is indicated where this estimation was not possible, *i.e.* when total N <10.

| **Country** | **Locality** | **Year** | **Species** |  | **Allele frequencies (support limits)** | | | | | | | | | |
| --- | --- | --- | --- | --- | --- | --- | --- | --- | --- | --- | --- | --- | --- | --- |
|  |  |  |  |  | ***R^x^*** | |  | ***R^x^**** | |  | *S* |  | ***D*** | |
| Ivory Coast | Bouaké | 2012 | *coluzzii* |  | - |  |  | - |  |  | - |  | - |  |
|  |  |  | Hybrids |  | - |  |  | - |  |  | - |  | - |  |
|  |  |  | *gambiae* |  | 0 | (0-0.1) |  | 0.18 | (0.1-0.29) |  | 0 |  | 0.82 | (0.57-0.9) |
|  |  | 2016 | *coluzzii* |  | 0 | (0-0.31) |  | 0 | (0-0.05) |  | 0.69 |  | 0.31 | (0-0.49) |
|  |  |  | *gambiae* |  | 0.03 | (0-0.16) |  | 0.31 | (0.21-0.41) |  | 0.54 |  | 0.12 | (0-0.25) |
|  | Yopougon | 2012 | *coluzzii* |  | 0 | (0-0.18) |  | 0 | (0-0.02) |  | 0.85 |  | 0.15 | (0-0.22) |
|  |  | 2015 | *coluzzii* |  | 0.13 | (0.03-0.27) |  | 0 | (0-0.02) |  | 0.47 |  | 0.4 | (0.23-0.55) |
|  |  | 2016 | *coluzzii* |  | 0.13 | (0.03-0.27) |  | 0 | (0-0.02) |  | 0.60 |  | 0.27 | (0.11-0.41) |
|  | Yamoussoukro | 2012 | *coluzzii* |  | 0 | (0-0.2) |  | 0 | (0-0.02) |  | 0.68 |  | 0.32 | (0.1-0.43) |
|  |  |  | *gambiae* |  | - |  |  | - |  |  | - |  | - |  |
|  |  | 2015 | *coluzzii* |  | 0.14 | (0.03-0.29) |  | 0 | (0-0.02) |  | 0.53 |  | 0.33 | (0.16-0.49) |
|  |  |  | *gambiae* |  | - |  |  | - |  |  | - |  | - |  |
|  |  | 2016 | *coluzzii* |  | 0 | (0-0.22) |  | 0 | (0-0.02) |  | 0.5 |  | 0.5 | (0.26-0.63) |
|  |  |  | Hybrids |  | - |  |  | - |  |  | - |  | - |  |
|  |  |  | *gambiae* |  | 0 | (0-0.21) |  | 0.12 | (0.04-0.27) |  | 0.72 |  | 0.16 | (0-0.34) |
|  | Man | 2012 | *coluzzii* |  | 0 | (0-0.03) |  | 0 | (0-0.03) |  | 1 |  | 0 | (0-0.03) |
|  |  |  | Hybrids |  | - |  |  | - |  |  | - |  | - |  |
|  |  |  | *gambiae* |  | 0 | (0-0.3) |  | 0 | (0-0.08) |  | 0.85 |  | 0.15 | (0-0.34) |
|  |  | 2015 | *coluzzii* |  | 0 | (0-0.08) |  | 0 | (0-0.02) |  | 0.97 |  | 0.03 | (0-0.08) |
|  |  |  | *gambiae* |  | 0 | (0-0.21) |  | 0.13 | (0.03-0.29) |  | 0.77 |  | 0.1 | (0-0.29) |
|  | M'Bé | 2012 | *coluzzii* |  | 0 | (0-0.12) |  | 0 | (0-0.02) |  | 0.93 |  | 0.07 | (0-0.13) |
|  |  |  | *gambiae* |  | - |  |  | - |  |  | - |  | - |  |
|  |  | 2016 | *coluzzii* |  | 0 | (0-0.09) |  | 0 | (0-0.02) |  | 0.96 |  | 0.04 | (0-0.09) |
|  |  |  | *gambiae* |  | - |  |  | - |  |  | - |  | - |  |
| Bénin | Natitingou | 2014 | *gambiae* |  | 0 | (0-0.06) |  | 0.01 | (0-0.04) |  | 0.97 |  | 0.02 | (0-0.06) |
|  |  | 2015 | *gambiae* |  | 0.02 | (0-0.06) |  | 0.1 | (0.05-0.16) |  | 0.88 |  | 0 | (0-0.06) |
|  |  | 2017 | *coluzzii* |  | 0 | (0-0.05) |  | 0 | (0-0.05) |  | 1 |  | 0 | (0-0.05) |
|  |  |  | Hybrids |  | - |  |  | - |  |  | - |  | - |  |
|  |  |  | *gambiae* |  | 0.13 | (0.04-0.26) |  | 0.66 | (0.52-0.79) |  | 0.21 |  | 0 | (0-0.05) |
| Togo | Baguida | 2013 | *gambiae* |  | 0.14 | (0.05-0.27) |  | 0.77 | (0.64-0.86) |  | 0 |  | 0.09 | (0-0.14) |
|  |  | 2014 | *coluzzii* |  | - |  |  | - |  |  | - |  | - |  |
|  |  |  | Hybrids |  | - |  |  | - |  |  | - |  | - |  |
|  |  |  | *gambiae* |  | 0.14 | (0.05-0.24) |  | 0.72 | (0.62-0.82) |  | 0 |  | 0.14 | (0-0.19) |
|  |  | 2016 | gambiae |  | 0.06 | (0-0.19) |  | 0.89 | (0.75-0.97) |  | 0 |  | 0.05 | (0-0.1) |
|  |  | 2017 | *coluzzii* |  | - |  |  | - |  |  | - |  | - |  |
|  |  |  | *gambiae* |  | 0 | (0-0.12) |  | 0.86 | (0.73-0.92) |  | 0 |  | 0.14 | (0-0.21) |
